# Understanding why women seek abortions in the US

**DOI:** 10.1186/1472-6874-13-29

**Published:** 2013-07-05

**Authors:** M Antonia Biggs, Heather Gould, Diana Greene Foster

**Affiliations:** 1 Advancing New Standards in Reproductive Health, Bixby Center for Global Reproductive Health, University of California, San Francisco, Oakland, California, USA

**Keywords:** Abortion, Women’s health, Qualitative research

## Abstract

**Background:**

The current political climate with regards to abortion in the US, along with the economic recession may be affecting women’s reasons for seeking abortion, warranting a new investigation into the reasons why women seek abortion.

**Methods:**

Data for this study were drawn from baseline quantitative and qualitative data from the *Turnaway Study*, an ongoing, five-year, longitudinal study evaluating the health and socioeconomic consequences of receiving or being denied an abortion in the US. While the study has followed women for over two full years, it relies on the baseline data which were collected from 2008 through the end of 2010. The sample included 954 women from 30 abortion facilities across the US who responded to two open ended questions regarding the reasons why they wanted to terminate their pregnancy approximately one week after seeking an abortion.

**Results:**

Women’s reasons for seeking an abortion fell into 11 broad themes. The predominant themes identified as reasons for seeking abortion included financial reasons (40%), timing (36%), partner related reasons (31%), and the need to focus on other children (29%). Most women reported multiple reasons for seeking an abortion crossing over several themes (64%). Using mixed effects multivariate logistic regression analyses, we identified the social and demographic predictors of the predominant themes women gave for seeking an abortion.

**Conclusions:**

Study findings demonstrate that the reasons women seek abortion are complex and interrelated, similar to those found in previous studies. While some women stated only one factor that contributed to their desire to terminate their pregnancies, others pointed to a myriad of factors that, cumulatively, resulted in their seeking abortion. As indicated by the differences we observed among women’s reasons by individual characteristics, women seek abortion for reasons related to their circumstances, including their socioeconomic status, age, health, parity and marital status. It is important that policy makers consider women’s motivations for choosing abortion, as decisions to support or oppose such legislation could have profound effects on the health, socioeconomic outcomes and life trajectories of women facing unwanted pregnancies.

## Background

While the topic of abortion has long been the subject of fierce public and policy debate in the United States, an understanding of why women seek abortion has been largely missing from the discussion [1]. In an effort to maintain privacy, adhere to perceived social norms, and shield themselves from stigma, the majority of American women who have had abortions— approximately 1.21 million women per year [2]– do not publicly disclose their abortion experiences or engage in policy discussions as a represented group [3-5].

A review of several international and a handful of US qualitative and quantitative articles considered reasons for abortion among women in 26 “high-income” countries [6]. Of these, four studies (two primarily quantitative, one primarily qualitative and one that used mixed methods) were conducted in the US [7-10]. This review found that, despite methodological differences among the studies, a consistent picture of women’s reasons for abortion emerged, that could be encapsulated in three categories: 1) “Women-focused” reasons, such as those related to timing, the woman’s physical or mental health, or completed family size; 2) “Other-focused” reasons, such as those related to the intimate partner, the potential child, existing children, or the influences of other people, and 3) “Material” reasons, such as financial and housing limitations. These categories were not mutually exclusive; women in nearly all of the studies reported multiple reasons for their abortion.

The largest of the US studies included in the review, by Finer and colleagues [9], utilized data from a structured survey conducted in 2004 with 1,209 abortion patients across the US, as well as open-ended, in-depth interviews conducted with 38 patients from four facilities, nearly half of whom were in their second trimester of pregnancy. Quantitative data from this study were compared to survey data collected from nationally representative samples in 1987 [11,12] and 2000 [13]. The most commonly reported reasons for abortion in 2004 (selected from a researcher-generated list of possible reasons with write-in options for other reasons) were largely similar to those found in the 1987 study [11]. The top three reason categories cited in both studies were: 1) “Having a baby would dramatically change my life” (i.e., interfere with education, employment and ability to take care of existing children and other dependents) (74% in 2004 and 78% in 1987), 2) “I can’t afford a baby now” (e.g., unmarried, student, can’t afford childcare or basic needs) (73% in 2004 and 69% in 1987), and 3) “I don’t want to be a single mother or am having relationship problems” (48% in 2004 and 52% in 1987). A sizeable proportion of women in 2004 and 1987 also reported having completed their childbearing (38% and 28%), not being ready for a/another child (32% and 36%), and not wanting people to know they had sex or became pregnant (25% and 33%). Considering all of the reasons women reported, the authors observed that the reasons described by the majority of women (74%) signaled a sense of emotional and financial responsibility to individuals other than themselves, including existing or future children, and were multi-dimensional. Greater weeks of gestation were found to be related with citing concerns about fetal health as reasons for abortion. The authors did not examine associations between weeks of gestation with some of the other more frequently mentioned reasons for abortion.

While the US abortion rate appears to have stabilized after a national decline, this decline has been slower among low-income women and in certain states, suggesting possible disparities in access to effective contraceptive methods and/or economic challenges preventing women from feeling they are able to care for a child [2,13]. According to national estimates for 2005 and 2008, changes in the abortion rate varied by region, with the South and West seeing small declines, and the Northwest and Midwest seeing no change over that period [2].

Furthermore, the changing political climate and increasing restrictive legislation with regards to abortion in this country [14], in conjunction with the economic recession, may be affecting women’s reasons for seeking abortion, warranting a fresh investigation into these issues. This study builds upon and extends the small body of literature that documents US women’s reasons for abortion [6]. While two other papers using data from the Turnaway Study (see below) describe how women who indicate partner related reasons or reasons related to their own alcohol, tobacco and/or drug use, differ from those who do not mention these reasons [15,16] this study presents all of the reasons women from the Turnaway Study gave for seeking abortion, as described in their own words.

## Methods

### Ethics statement

This study was approved by the University of California, San Francisco, Committee on Human Research. Written and oral consent was obtained from all participants.

### Study design

Data for this study were drawn from baseline quantitative and qualitative data from the Turnaway Study, an ongoing, five-year, longitudinal study evaluating the health and socioeconomic consequences of receiving or being denied an abortion in the US. While the study has followed women for two full years, this analysis relies on the baseline data which were collected from 2008 through the end of 2011. The study design, recruitment and research methods and some findings from this study have been published elsewhere [15,17-19]. This study overcomes several limitations of previous studies on this topic. Most importantly, we interviewed a large sample of both adult and adolescent women, including many women who sought abortions at later gestations of pregnancy. We asked women about their reasons for abortion using an open-ended question, rather than relying on a checklist of researcher-generated reasons.

This paper draws on baseline data from interviews conducted one week after receiving or being denied an abortion at the recruitment facility.

### Recruitment

Women seeking abortion care at 30 US facilities (abortion clinics, other clinics and hospitals) between January 2008 and December 2010 were recruited to participate in the study. Facilities were identified using the National Abortion Federation membership directory, as well as through professional contacts in the abortion research community. While the gestational limits of the recruitment facilities varied (from 10 weeks to the end of the second trimester), they each had the latest gestational limit for providing abortion of any facility within 150 miles. These sites were selected because we thought that women denied an abortion would be unlikely to get one elsewhere. The facilities performed an average of 2,400 abortions annually (range 440–8,000) and were located in 21 states throughout the US representing every US region [17].

Abortion patients were eligible to participate in the study if they were English- or Spanish-speaking, aged 15 years or older, had no fetal diagnoses or demise, and were within the gestational age range of one of three study groups. At each facility, a designated point person was trained by Turnaway study researchers to oversee and conduct site recruitment activities. After assessing potential participants’ eligibility based on their age, language and gestational age, the facility point person or facility staff briefly informed potential participants about the study. Participants were usually approached in a private exam room after receiving an ultrasound and were told that the purpose of the study was to learn more about how unintended pregnancy affects women’s lives. Participants who expressed willingness to learn more about the study were led to a private location within the clinic, where they were given additional study information, the informed consent documents, and human subjects’ Bill of Rights^a^.

Facility staff then connected interested prospective participants to Turnaway study researchers by telephone. Facility staff dialed and introduced participant by first name then passed the phone to the woman to speak with the interviewer. During the recruitment call, research staff explained the study in greater detail, screened for eligibility and obtained informed consent. After verbally agreeing to participate, each woman signed a written consent form, which was faxed by facility staff to a private, dedicated fax machine in the UCSF Project Director’s office. Parental consent was obtained from women under the age of 18 living in states where parental notification or consent for an abortion is required. In states without parental involvement laws, women under the age of 18 were screened for their ability to understand the risks and benefits of the study and, those who were determined to be able provided informed consent on their own behalf. For all patients who completed the recruitment call and consented to enroll, Turnaway study researchers scheduled their first telephone interview to take place eight days later. These baseline interviews lasted approximately 40 minutes. The study is ongoing, with follow-up phone interviews being conducted every six months for five years.

All interviewers were female, fluent in English and/or Spanish, and experienced in reproductive health research and interviewing techniques. The interviewer training covered general interviewing guidelines, handling sensitive issues, confidentiality, data collection protocols, question-by-question reviews of both English and Spanish versions of the interview guide, role playing, and record-keeping. During the data collection period, research staff worked closely with the interviewers to ensure data quality. Quality assurance strategies included making sure that interviewers understood the meaning of every question, how to ask the question and how to record answers, observation of live interviews, monitoring the data for missing values, and periodic inter-rater reliability tests. All data from the interviews were entered manually. The interviewers simultaneously collected and entered data into a password-protected, computer database using CASES (Computer Assisted Survey Execution System). Qualitative responses in Spanish were translated to English by bilingual research staff. Women were mailed a $50 gift card for a major retail store after completing each interview.

### Participants

Overall, 37.5% of eligible women agreed to complete semi-annual telephone interviews for a period of five years. For the purposes of the larger study, participants were recruited into three distinct study groups: women who were denied an abortion because they were just over the pregnancy gestational age limit for the clinic (n=231), women who received an abortion and were just under the gestational age limit (n=452), and women who received a first trimester procedure (n=273). For the purposes of this analysis, all three groups are combined and analyzed by gestational age.

### Measures

The structured interview guide included questions on participant socio-demographic characteristics, experiences becoming pregnant, pregnancy planning, and the abortion decision-making process. The interview guide and study protocols were all first pilot tested among 64 women receiving or being denied an abortion at a local abortion facility.

#### Demographic characteristics

We examine age, race/ethnicity (White, Black, Hispanic/Latina and other), education (more than high school versus high school graduation or less than high school), whether they received public assistance (i.e. Women Infant and Children (WIC), food stamps, disability payments, or Temporary Assistance for Needy Families (TANF)) in the past month and employment status (part/full time versus not employed).

#### Pregnancy-related characteristics

We also considered parity, and gestational age at recruitment (13 weeks or less, 14 to 19 weeks, and 20 weeks or more). Pregnancy intentions were measured with the London Measure of Unplanned Pregnancy. The London Measure is a validated measure of pregnancy intentions that assesses contraceptive use, intentions to become pregnant, extent to which women wanted to become pregnant and partner interest in becoming pregnant in the month before becoming pregnant as well as changes women may have made in preparation for pregnancy and women’s perceptions of the timing of the pregnancy [20]. It is a continuous scale ranging from 1–12, with 0–3 indicating unplanned pregnancies, 4–9 ambivalent pregnancies and 10–12 planned pregnancies.

#### Health care and health

*“*Has healthcare provider” was a dichotomous variable defined as having a doctor or nurse practitioner one usually goes to when sick or wanting health advice. Self-rated health is a dichotomous variable of rating health prior to pregnancy as good or very good versus fair, poor or very poor. History of depression or anxiety diagnosis is a dichotomous variable indicating whether the participant has ever been told by a health professional if she suffers from a major depressive or anxiety disorder.

#### Reasons for abortion

All participants were asked two open-ended questions about their reasons for seeking an abortion. The first question asked “What are the reasons that you decided to have an abortion?” followed by a prompt asking for any other reasons until the respondent says that is all. The second questions asked “What would you say was the *main reason* you decided to have an abortion?” Generally participants were not able to narrow their answers to one reason and sometimes even gave additional reasons to this last question making it difficult to discern a “main” reason. Therefore, the answers to both questions were combined to identify all reasons given by respondents for seeking abortion.

### Data analysis

#### Qualitative analysis

The analytic team was comprised of two of the study authors. A non-hierarchical list of themes was generated and agreed upon by both researchers after reviewing an initial 100 responses. The next set of 100 responses was coded using the agreed upon themes and were revised iteratively, as appropriate. The list of themes was finalized after review of all responses. Once the final set of themes was generated, both researchers recoded all the responses until reaching consensus on all items. Occasionally the underlying reasons that motivated a particular response were not evident. For example some women may have responded that they sought abortion due to “bad timing”, which may have been due to a number of factors (e.g. being financially unprepared or not having found the right partner) but unless these underlying reasons were explicitly stated, her reason was coded only as “bad timing.” Often the reasons were interrelated with other reasons, (e.g. “bad timing because I’m unemployed”) in which case the response was coded under all themes mentioned (e.g. “bad timing” and “unemployed”). Respondents could also be coded under multiple subthemes within an overarching theme (e.g. “unemployed and don’t want government assistance.” All coding was done in Excel.

#### Quantitative analysis

Once all of the codes were finalized, the reasons for abortion were analyzed quantitatively using Stata Version 12. Multivariable mixed effects logistic regression was used to assess the characteristics associated with having higher odds of reporting each of the major themes as a reason for seeking abortion. Continuous predictors included age, pregnancy intentions and parity. Dichotomous predictors included high school education and above (yes/no), employed (yes/no), has health care provider (yes/no), history of depression or anxiety (yes/no), and rates health as good/very good (yes/no). Additional categorical predictors included a four-part race variable, a three-part marital status variable, and a three-part gestational age variable. Our quantitative analysis approach accounted for clustering by recruitment site.

## Results

### Description of the sample

Two women did not answer either question on reason for seeking an abortion, leaving a final sample of 954. A description of study participants is presented in Table 1. Approximate 37% of participants were white, 36% between the ages of 20 and 24 (36%), and 38% were nulliparous. The majority were single and never married (79%), had less than a high school education (53%) and enough money to meet basic living needs (60%).

**Table 1:** Participant characteristics (n=954)

**Participant characteristics**	**N**	**%**
**Race/ethnicity**		
White	353	37
Black	281	29
Hispanic/Latina	199	21
Other	121	13
**Age group**		
15–19^a^	173	18
20–24	345	36
25–34	365	38
35–46	71	7
**Marital status**		
single, never married	753	79
Married	86	9
separated, divorced, widowed	115	12
More than High School education	450	47
Enough money in past month to meet basic needs	569	60
Received public assistance in past month	428	45
Employed	507	53
**Gestational age at interview**		
<=13 weeks	393	41
14-19 weeks	136	14
20+ weeks	425	45
**Pregnancy intentions (mean)**	954	2.7
**Parity (mean)**	943	1.27
Nulliparous	359	38
baby under one	101	11
1+ previous births, no new baby	233	24
2+ previous births, no new baby	261	27
Has a health care provider	422	45
History of depression or anxiety diagnosis	260	27
Self-rated health good/very good	775	81

^a^This age category includes one participant aged 14 who was recruited early in the study before the minimum enrollment age was changed to 15.

### Reasons for abortion

Women gave a wide range of responses to explain why they had chosen abortion. The reasons were comprised of 35 themes which were categorized under a final set of 11 overarching themes (Table 2). While most women gave reasons that fell under one (36%) or two (29%) themes, 13% mentioned four or more themes. Many women reported multiple reasons for seeking an abortion crossing over several themes. As one 21 year-old woman describes, “*This is how I described it [my reasons for abortion] to my doctor 'social, economic’, I had a whole list, I don’t feel like I could raise a child right now and give the child what it deserves.*”A 19-year old explains *“[There are] so many of them [reasons]. I already have one baby, money wise, my relationship with the father of my first baby, relationship with my mom, school.*” A 27-year old enumerates the reasons that brought her to the decision to have an abortion “*My relationship is newer and we wanted to wait. I don’t have a job, I have some debt, I want to finish school and I honestly am not in the physical shape that I would want to be to start out a pregnancy*.”

**Table 2:** Major themes and reasons women gave for seeking abortion (n=954)

		**Freq.**	**Percent**
**Not financially prepared**		**386**	**40%**
General financial		365	38%
Unemployed/underemployed		41	4%
Uninsured or can't get welfare		6	0.6%
Don't want government assistance		4	0.4%
**Not the right time for a baby**		**347**	**36%**
Bad timing/not ready/unplanned		321	34%
Too busy/not enough time		17	2%
Too old		16	2%
**Partner related reasons**		**298**	**31%**
Relationship is bad, poor and/or new		89	9%
Respondent wants to be married first/not a single mom		80	8%
Partner is not supportive		77	8%
Partner is wrong guy		61	6%
Partner does not want baby		29	3%
Partner is abusive		24	3%
**Need to focus on other children**		**275**	**29%**
Too soon after having had a child/busy enough with current children/have enough children right now		239	25%
Concern for other children she is rearing		51	5%
**Interferes with future opportunities**		**194**	**20%**
Interferes with educational plans		132	14%
Interferes with vocational plans		63	7%
Want better life for self/don't want to limit future opportunities		49	5%
**Not emotionally or mentally prepared**		**180**	**19%**
**Health related reasons**		**114**	**12%**
Concern for her own health		59	6%
Concern for the health of the fetus		51	5%
Drug, tobacco, or alcohol use		46	5%
Prescription drug (not illicit) or contraceptive use		14	1.5%
**Want a better life for the baby than she could provide**		**119**	**12%**
Want better life for baby		67	7%
Living or housing context not suitable for baby		46	5%
Lack of childcare or help from family to care for baby		13	1.4%
Don't want her children to have a childhood like hers		5	0.5%
**Not independent or mature enough for a baby**		**64**	**7%**
Too young or immature		47	5%
Can't take care of self		12	1.3%
Too dependent on parents or others right now		9	0.9%
**Influences from family or friends**		**48**	**5%**
Would have a negative impact on family or friends		22	2%
Don't want others to know/worried others would judge		19	2%
Pressure from family or friends		11	1.2%
**Don't want a baby or place baby for adoption**		**38**	**4%**
Don't want a baby or don't want any children		33	3%
Don't want adoption		7	0.7%
**Other**		**11**	**1.2%**
**Total**		**954**	**100%**

Note: Respondents gave reasons under multiple themes and subthemes.

#### Financial reasons

A financial reason (40%) was the most frequently mentioned theme. Six percent of women mentioned this as their only reason for seeking abortion. Most women (38%) cited general financial concerns which included responses such as “*financial problems*,” “*don’t have the means*,” *“It all boils down to money*” and “*can’t afford to support a child*.” As one unemployed 42-year-old woman with a monthly household income of a little over $1,000 describes *“[It was] all financial, me not having a job, living off death benefits, dealing with my 14 year old son. I didn't have money to buy a baby spoon.*”

A small proportion of women (4%) stated that lack of employment or underemployment was a reason for seeking an abortion. A 28-year old college educated woman, receiving $1,750 a month in government assistance, looking for work, and living alone with her two children while her husband was away in the Air Force explains “*[My husband and I] haven't had jobs in awhile and I don't want to go back to living with other people. If we had another child it would be undue burden on our financial situation.*” Six (0.6%) women stated that their lack of insurance and/or inability to get government assistance contributed to their desire to terminate their pregnancies.

*“I’m unemployed, no health insurance, and could not qualify for any government-assisted aid, and even if my fiancé decided to hurry up and get married, I still wouldn't have been covered under his health insurance for that.”--*32-year-old, in school full-time.

Four respondents (0.4%) said that their desire to have an abortion stemmed from their inability to provide for the child without relying on government assistance. *“I don't have enough money to support a child and I don't want to have to get support from the government.*”

#### Not the right time for a baby

Over one third (36%) of respondents stated reasons related to timing. Many women (34%) used phrasing such as “*I wasn’t ready*”, and “*wasn’t the right time.*” A 21-year old pointed to a number of reasons why she felt the timing of her pregnancy was wrong “*Mainly I didn't feel like I was ready yet - didn't feel financially, emotionally ready. Due date was at the same time as my externship at school. Entering the workforce with a newborn would be difficult - I just wasn't ready yet*.” A small proportion of women described not having enough time or feeling too busy to have a baby (2%). A 25-year old looking for work, already raising a child, and who reported “rarely” having enough money to meet her basic living needs explains how she has “*So many things going on now-physically,emotionally, financially, pretty busy and can't handle anymore right now.*” Similarly, a 19-year old describes how she “*didn't have time to go to the doctor to make sure everything is OK like I wanted to. So busy with school and work I felt it [having an abortion] would be the right thing to do until I really have time to have one [a child].*” Fewwomen described being too old to have a baby (2%). A 43-year old illustrates how timing and her age are the primary reasons for seeking abortion “*Because I'm too old to have a child. It's like starting over and my nerves are bad. My son…he's going to be 2b0 next month and I don't want to start over. It's just bad timing*.”

#### Partner-related reasons

Nearly one third (31%) of respondents gave partner-related reasons for seeking an abortion. Six percent mentioned partners as their only reason for seeking abortion. Partner related reasons included not having a “good” or stable relationship with the father of the baby (9%), wanting to be married first (8%), not having a supportive partner (8%), being with the “wrong guy” (6%), having a partner who does not want the baby (3%), and having an abusive partner (3%). For a more extensive analysis of partner-related reasons for seeking an abortion see Chibber et al. [17].

#### Need to focus on other children

The need to focus on other children was a common theme, mentioned by 29% of women. Six percent of women mentioned only this theme. The majority of these reasons (67%) were related to feeling overextended with current children “*I already had 2 kids and it would be really overwhelming. It's kind of hard to raise 2 kids by yourself*,” that the pregnancy was too soon after a previous child “*I have a 3-month-old already. If I had had that baby, he wouldn't even be one [year old by the time the baby came]*”, or simply not wanting any more children *“I just felt inadequate-I have a teenager and 2 pre-teens and I couldn't see starting over again.”* A small proportion (5%) of women felt that having a baby at this time would have an adverse impact on her other children. *“I already have 5 kids; their quality of life would go down if I had another.*” A 31-year-old with three children spoke of the need to focus on her sick child as a reason for seeking abortion *“My son was diagnosed with cancer. His treatment requires driving 10 hours and now we found out we need to go to New York for some of his treatment. The stress of that and that he relies on me.*”

#### A new baby would interfere with future opportunities

One in five women (20%) reported that they chose abortion because they felt a baby at this time would interfere with their future goals and opportunities in general (5%) or, more specifically, with school (14%) or career plans (7%). Usually the reasons were related to the perceived difficulty of continuing to advance educational or career goals while raising a baby: *“I didn't think I'd be able to support a baby and go to college and have a job.*” states an 18-year old respondent in high school. A 21-year-old woman in college with no children explains that she “*Still want[s] to be able to do things like have a good job, finish school, and be stable.*” Similarly*,* a 26-year old desiring to go back to college explains “*I wanted to finish school. I'd been waiting a while to get into the bachelor's program and I finally got it.*” Another woman explains “*I feel like I need to put myself first and get through college and support myself.*” As a 21-one-year old seeking a college degree points out, *“I’m trying to graduate from college and I’m going to cooking school in August and I have a lot of things going for me and I can’t take care of a kid by myself*.” Others spoke to the inability to take time off work to raise the child.” A 21-one-year old holding two part-time jobs and raising two children states: *“I wouldn't be able to take the time off work. My work doesn't offer maternity leave and I have to work [to afford to live] here. If I took time off I would lose my job so there's just no way.”*

Some women, particularly younger women, expressed the feeling that having a baby at this time would negatively impact multiple aspects of their future lives.

*“It is hard to get in school. If I had the baby it would be tough to do school work, thinking about my future. I know that I wouldn't be able to do what I want to do. I still want to be free and have my youth. I don't want to have it all gone because of one experience. I still want to study abroad. I don't want to ruin that.” --*20-year-old in college with no children

#### Not emotionally or mentally prepared

Nineteen percent of respondents (19%) described feeling emotionally or mentally unprepared to raise a child at this time. Respondents in this category were characterized by a feeling of exasperation and an inability to continue the pregnancy— *“I can't go through it”*, “*I just felt inadequate*”— or feeling a lack of mental strength to have the baby— *“[I am] not mentally stable to take that on”, “emotionally, I couldn't take care of another baby,*” and *“I couldn’t handle it.”* A 19-year old mother reporting a history of depression and physical abuse describes seeking an abortion because, “*I have a lot of problems-serious problems and so I'm not prepared for another baby.*” Another woman explained her rationale for seeking abortion, “*I would say a mental reason, in the sense that it would really be a burden because then I would have to watch three, my hands are already full.”*

#### Health-related reasons

Twelve percent of respondents (12%) mentioned health-related reasons ranging from concern for her own health (6%), health of the fetus (5%), drug, tobacco, or alcohol use (5%), and/or non-illicit prescription drug or birth control use (1%). Maternal health concerns included physical health issues that would be exacerbated by the pregnancy or due to the pregnancy itself, “*My bad back and diabetes, I don't think the baby would have been healthy. I don't think I would have been able to carry it to term” *as well as mental health concerns. Five percent of women (5%) chose abortion because they were concerned about the effects of their drug and/or alcohol use on the health of the fetus or on their ability to raise the child. For a more extensive analysis of substance use as reasons for seeking an abortion see Roberts et al. [16]. Other women (5%) voiced concern for the health of the fetus because they had been using contraceptives (n=4), psychotropic drugs (n=3) or medications (such as antibiotics, blood thinners, and narcotics) to treat other health conditions (n=7). As one woman explains, she and her partner chose abortion “*because I had been doing drinking and the medication I’m on for bipolar disorder is known to cause birth defects and we decided it’s akin to child abuse if you know you’re bringing your child into the world with a higher risk for things.*”

#### Want a better life for the baby than she could provide

Twelve percent of women gave reasons for choosing abortion related to their desire to give the child a better life than she could provide. Responses related to generally wanting to give the child a better life (7%) were characterized by a concern for the child *“I'm afraid my kid will be suffering in this world”* and “*wouldn't have been good for me or the child,”*or a feeling of inadequacy to parent the child: *“I can't take care of a kid because I can barely take care of myself and I don't want to bring a child into the world when I'm unmarried and not ready.*” As reflected in this previous quote, sometimes statements stemmed from a desire for the baby to have a father, or the feeling that the father of the baby was not suitable. *“I didn't want to do it by myself. I couldn't and the man was abusive and horrible… I didn't want my kid to grow up with a father like that (knowing his father had left).*” For one woman, the decision to terminate her pregnancy was a moral one. *“I've been unemployed it’s not a decision I can face morally without being able to raise it properly. An abortion was the best option.*”

Approximately 5% of respondents explained that their living or housing context was not suitable for a baby and mentioned this as one of the reasons they chose abortion. According to a 22-year old who described herself as being unable to work, on welfare, and rarely having enough money to meet basic living needs: *“My mom pays my rent for me and where I live I can't have kids. I can't get anyone to rent to me because I have had an eviction and haven't had a steady job.*”

While never mentioned as the only reason for choosing abortion, 13 respondents said that lack of help to care for the baby was one reason they chose abortion. Responses included *“I wouldn't have a babysitter for school,” “family isn't close by to help”,* and *“My grandma passed away and she was the one who was going to help.”*Another subcategory of this theme included choosing abortion because of the desire not to repeat their childhood (n=5). An 18-year old who frequently smoked marijuana explained that she chose abortion *“Because I did do drugs and my mom used drugs with me and my sister and I swore to myself I wouldn't bring a child into this world like that.*” Another respondent in her teens and who had a history of physical and sexual abuse and neglect remarked *“my childhood was less than awesome, if I do have a child I want to give it the best possible life that I can and I am not in a place to do that right now.*”

#### Lack of maturity or independence

Less than 7% of women explained that their reliance on others or lack of maturity was a reason for choosing abortion. Some women felt they were too young (5%), unable to take care of themselves (1%), or too reliant on others to raise this baby (1%). *“I'm not grown up enough to take care of another person. I can't take care of myself yet, let alone another person. I wouldn't want to bring a baby into this world with parents who aren't ready to be parents.*”

#### Influences from friends and/or family

Around 5% of women described a concern for, or influences from family or friends as a reason for seeking abortion. Two percent feared that having a baby would negatively impact their family or friends *“It would have been a strain on my family*” and a similar proportion (2%) didn’t want others to know about their pregnancy or feared judgment or reaction from others. A 19-year old explains that the reason she chose abortion was because *“I was scared to go to my parents*.” Another woman feared what the family would think about her having a biracial child. A small minority reported influences or pressure from family or friends (n=11) as a reason for seeking abortion. *“Because my mother convinced me to get one,*” explains one 17-year old. A 23-year old describes her rationale for seeking abortion *“because my dad thinks I should finish school first, not financially ready for a baby, gonna have to move out when I have the baby.*” Similarly, a 25-year old explained that she wanted an abortion because of, *“the negative feedback I was getting from my family.*”

#### Don’t want a baby or place baby for adoption

Four percent (4%) of women gave reasons falling under the theme not wanting a baby or not wanting to place a baby for adoption. Three percent (3%) explained succinctly that they do not want a baby or don’t want children *“I just didn't want any kids”, “It [a baby] is something I just didn't want.”* A small number (n=7) mentioned adoption was not an option for them. As one 25-year old describes *“We are not really sure if we ever want kids. I don't think that I would be strong enough to give it up for adoption.*” Another respondent states that *“adoption isn't an option for me-so it was kind of a no brainer decision.*”

#### Other reasons

Eleven women (1%) gave other reasons for seeking abortion that didn’t easily fall into one of the major themes, including going through legal issues (n=3) and fear of giving birth (n=2).

### Factors related to reasons for abortion

Using mixed effects multivariate logistic regression analyses, we examined the social and demographic predictors of the predominant themes women gave for seeking an abortion (Table 3). Significant predictors of reporting financial reasons for seeking an abortion included marital status, education level, and not having enough money to meet basic living needs. Women who gave financial reasons for seeking an abortion were more likely to have a higher level of education [Odds Ratio (OR) 1.41, 95% Confidence Interval (CI), 1.05-1.90], less likely to be separated, divorced or widowed (OR, 0.54, CI, 0.34-0.86) than to be single/never married, and less likely to have enough money to meet basic needs (OR 0.54 CI, 0.41-0.72). Approximately 82% of women who reported this as a reason were single/never married.

**Table 3:** Multivariate mixed effects logistic analyses predicting reasons for abortion

**Participant characteristics**	**Not financially prepared**			**Need to focus on other children now**			**Not the right time for baby**			**Partner related reason**			**Interferes with future opportunities**			**Not emotionally or mentally prepared**			**Health related reasons**			**Want a better life for the baby than she could provide**			**Not independent or mature enough to raise baby**			**Influences from family or friends**			**Don’t want a baby or place baby for adoption**		
	**OR**	**95% CI**		**OR**	**95% CI**		**OR**	**95% CI**		**OR**	**95% CI**		**OR**	**95% CI**		**OR**	**95% CI**		**OR**	**95% CI**		**OR**	**95% CI**		**OR**	**95% CI**		**OR**	**95% CI**		**OR**	**95% CI**	
**Race/ethnicity**																																	
*White (reference)*																																	
*Black*	0.74	0.51	1.06	1.17	0.76	1.80	0.81	0.56	1.18	0.66*	0.45	0.99	1.28	0.82	2.00	0.47***	0.29	0.75	0.62	0.33	1.14	0.60	0.35	1.03	0.45	0.20	1.01	0.76	0.31	1.88	1.16	0.47	2.85
*Hispanic/Latina*	0.77	0.52	1.13	0.93	0.58	1.49	0.94	0.64	1.40	0.99	0.65	1.49	0.92	0.56	1.51	0.80	0.50	1.27	0.90	0.49	1.64	0.48	0.26	0.89	0.63	0.29	1.37	1.36	0.57	3.25	1.27	0.52	3.11
*Other*	0.93	0.60	1.46	1.15	0.67	1.95	0.84	0.52	1.35	0.92	0.57	1.49	1.26	0.71	2.22	0.58	0.33	1.04	0.83	0.42	1.63	0.63	0.32	1.24	0.47	0.16	1.37	1.45	0.54	3.89	0.49	0.11	2.28
**Age**	1.01	0.98	1.04	0.97	0.94	1.01	1.02	0.99	1.05	1.03*	1.00	1.07	0.94***	0.90	0.98	0.98	0.94	1.02	1.10***	1.05	1.14	1.00	0.95	1.04	0.83***	0.75	0.92	0.87**	0.78	0.96	1.06	0.99	1.13
**Marital status**																									Not included due to collinearity								
*Single, never married (ref)*																																	
*Married*	0.65	0.39	1.07	1.09	0.63	1.88	0.75	0.44	1.27	1.15	0.68	1.95	0.74	0.36	1.50	1.05	0.57	1.93	1.29	0.65	2.56	1.03	0.47	2.25		1.03	0.22	4.81	1.61	0.55	4.72		
*Separated/divorced/widowed*	0.54*	0.34	0.86	1.10	0.66	1.83	0.90	0.56	1.46	2.22***	1.40	3.53	1.00	0.53	1.88	0.60	0.32	1.11	1.13	0.61	2.11	1.01	0.50	2.06		0.98	0.20	4.85	0.68	0.18	2.55		
**More than high school education**	1.41*	1.05	1.90	0.96	0.67	1.36	0.92	0.68	1.25	1.36	0.99	1.87	2.43***	1.66	3.56	1.14	0.79	1.66	1.11	0.69	1.77	1.61*	1.03	2.51	0.99	0.51	1.94	1.46	0.71	2.97	0.70	0.33	1.49
**Enough money in past month to meet basic needs**	0.54***	0.41	0.72	0.83	0.59	1.16	1.26	0.94	1.70	0.82	0.60	1.11	1.31	0.90	1.90	0.55***	0.38	0.78	1.23	0.78	1.94	0.79	0.52	1.21	0.68	0.37	1.26	1.08	0.54	2.16	1.19	0.57	2.49
**Employed**	1.06	0.80	1.42	0.99	0.70	1.39	1.29	0.96	1.73	1.24	0.91	1.68	0.98	0.68	1.40	0.70	0.49	1.01	0.50***	0.32	0.80	0.67	0.44	1.02	1.00	0.54	1.85	0.69	0.35	1.36	1.28	0.62	2.64
**Gestational age at interview**																																	
*<=13 weeks (reference)*																																	
*14-19 weeks*	0.97	0.63	1.48	0.83	0.51	1.35	1.06	0.69	1.63	0.71	0.45	1.13	1.04	0.62	1.75	1.37	0.83	2.26	1.23	0.65	2.35	1.21	0.65	2.25	1.69	0.66	4.37	1.18	0.44	3.18	1.97	0.73	5.30
*20+ weeks*	1.32	0.97	1.78	0.72	0.50	1.04	0.93	0.68	1.27	0.72	0.51	1.00	0.91	0.62	1.33	0.86	0.58	1.27	1.13	0.70	1.85	1.07	0.68	1.68	1.94	0.94	3.99	1.12	0.51	2.42	1.63	0.74	3.59
**Pregnancy intentions**	0.95	0.87	1.03	0.79***	0.71	0.88	0.85***	0.78	0.94	1.11*	1.01	1.21	0.89*	0.80	0.99	1.01	0.91	1.12	1.10	0.98	1.24	1.01	0.89	1.14	0.87	0.71	1.05	1.20*	1.01	1.42	0.77*	0.60	0.99
**Parity**	1.11	0.98	1.25	2.31***	1.97	2.72	0.71***	0.61	0.82	0.78***	0.67	0.90	0.83	0.69	1.00	0.94	0.80	1.11	0.84	0.70	1.02	0.65***	0.52	0.83	0.38***	0.22	0.67	0.71	0.45	1.11	0.67*	0.46	0.96
**Has a health care provider**	0.92	0.70	1.22	1.18	0.85	1.64	1.17	0.88	1.55	1.06	0.79	1.43	1.41	1.00	1.99	1.16	0.82	1.64	0.99	0.64	1.53	0.63*	0.42	0.96	0.85	0.47	1.53	1.59	0.84	3.00	0.91	0.46	1.81
**History of a depression or anxiety diagnosis**	1.12	0.81	1.55	0.83	0.57	1.23	0.79	0.56	1.11	0.91	0.65	1.29	0.66	0.43	1.03	1.06	0.72	1.57	3.29***	2.07	5.23	1.37	0.87	2.18	1.61	0.82	3.17	0.77	0.33	1.78	2.10	0.99	4.46
**Self-rated health good/very good**	1.02	0.72	1.45	1.18	0.77	1.78	0.95	0.66	1.37	1.02	0.70	1.49	1.81*	1.08	3.04	0.74	0.48	1.13	0.61*	0.37	0.99	1.03	0.61	1.75	2.21	0.75	6.50	2.38	0.70	8.07	1.28	0.50	3.23

*p<.05; **p<.01; ***p<.001; *OR* Odds Ratios; CI=95% Confidence Intervals.

Women who reported reasons related to the need to focus on other children now were significantly more likely to have a lower pregnancy intentions score (OR 0.79, CI 0.71-0.88), and, to have a greater number of children (OR 2.31, CI 1.97-2.72). All women who reported this as a reason had one or more children.

Women who reported that this is not the right time for a baby as a reason for seeking abortion had a lower pregnancy intentions score (OR 0.86, CI 0.78-0.94) and lower parity (OR 0.71, CI 0.61-0.82). Over half (51%) of women who reported this as a reason had no children.

Women who gave partner related reasons were significantly more likely to be African American (OR 0.66, CI 0.45-0.99) and to have higher parity (OR, 0.78, CI 0.67-0.90). Older women (OR 1.03, 1.0-1.07), women who were separated, divorced or widowed (OR 2.22, CI 1.40-3.53), and women with higher pregnancy intention scores (OR 1.11, CI 1.01-1.21), had increased odds of giving partner related reasons.

Women who chose abortion because they felt having a baby would interfere with her future plans were more likely to be younger (OR 0.94, CI 0.90-0.98), to have more than a high school education (OR 2.43, CI 1.66-3.56), self-rated good health (OR 1.81, CI 1.08-3.04), and lower scores on the pregnancy intentions scale (OR 0.89, CI 0.80-0.99). Among those who reported this as a reason, over half (52%) were in college or getting their Associates or technical degree.

Predictors of reporting being emotionally or mentally unprepared as a reason for seeking abortion included race/ethnicity and having enough money to meet basic living needs. Women who were African American (OR 0.47, CI 0.29-0.75) were less likely than white women to report this as a reason. Women who reported having sufficient money to meet basic needs (OR 0.55, CI 0.38-0.78) were at a reduced odds of reporting this as a reason for seeking abortion.

Women with a history of depression or anxiety (OR 3.29, CI 2.07-5.23) had sharply elevated odds of mentioning physical or mental health factors as reasons for seeking abortion. Women who rated their health as good (OR 0.61, CI 0.37-0.99) and were employed (OR 0.50, CI 0.32-0.80) had reduced odds of mentioning physical or mental health reasons for seeking abortion.

Women who chose abortion because they wanted to give the baby a better life than they could provide were significantly more likely to have more than a high school education (OR 1.61, CI 1.0-2.5), have lower parity (OR 0.65, CI 0.5-0.8), and to lack a usual health care provider (OR 0.63, CI 0.4-1.0). Over half of women who gave this as a reason were nulliparous (55%).

Women who gave lack of independence or immaturity as a reason for seeking abortion were more likely to be younger (OR 0.83, CI 0.7-0.9) and lower parity (OR 0.38, CI 0.2-0.7). All women who gave this reason were under age 31, 48% were in their teens and 83% were nulliparous. Marital status was excluded in the model because of problems with collinearity with the outcome. Nearly all (97%) women who gave this as a reason were single/never married.

Reporting influences from friends and family as a reason for seeking abortion was significantly predicted by age and pregnancy intentions. Women who report this reason were more likely to be younger (OR 0.87, CI 0.8-1.0) and to have a higher pregnancy intentions score (OR 1.20, CI 1.0-1.4). Over three quarters (85%) of women who gave this as a reason were ages 24 and under. Their average pregnancy intentions score was higher when compared to women giving other reasons (3.2 vs. 2.7, p=.03).

The two significant predictors of “don’t want a baby or place baby for adoption” were lower parity (OR 0.67, CI 0.46-0.96) and a lower pregnancy intentions score (OR 0.77, CI 0.60-0.99). Over two thirds (68%) who reported this reason were nulliparous.

## Discussion

The findings from this study demonstrate that the reasons women seek abortion are complex and interrelated. Unlike other studies [6], this study asked women entirely open-ended questions regarding the reasons they sought to terminate their pregnancies, ensuring that all women’s reasons could be fully captured. This methodology enabled us to get a wide range of responses that otherwise would not have been gathered. While some women stated only one factor that contributed to their desire to terminate their pregnancies, others pointed to a myriad of factors that, cumulatively, resulted in their seeking an abortion.

As indicated by the differences we observed among women’s reasons by individual characteristics, women seek abortion due to their unique circumstances, including their socioeconomic status, age, health, parity and marital status. Even with changes in the climate surrounding abortion and the shifting demographics of the women having abortions, the predominant reasons women gave for seeking abortion reflected those of previous studies [6]. Reasons related to timing, partners, and concerns for the ability to support the child and other dependents financially and emotionally were the most common reasons women gave for seeking an abortion, suggesting that abortion is often a decision driven by women’s concerns for current and future children, family, as well as existing commitments and responsibilities. Some women held the belief that her unborn child deserves to be raised under better circumstances than she can provide at this time; in an environment where the child is financially secure and part of a stable and loving family. This intersection between abortion and motherhood is described qualitatively in a study by Jones and colleagues where women indicate that their abortion decisions are influenced by the idea that children deserve “ideal conditions of motherhood” [21]. Some women also seem to have internalized gendered norms that value women as self-denying and always thinking in the best interest of her children, over making self-interested decisions. Experiences of stigma, fear of experiencing stigma, or internalized stigma around her abortion may have prompted women to give more socially desirable responses to make her appear or feel selfless, to justify her abortion decision. Other studies have reported abortion-seeking women’s fear of being judged as having made a selfish decision [22]. At the same time, some of the women seeking abortion in this study were aiming to secure themselves a better life and future- chances for a better job and a good education. These women may be more stigmatized than the former since they don’t fall into a discourse of the selfless and all-sacrificing woman. In an effort not to further contribute to the abortion stigma in our culture, we must be careful not to use women’s reasons for abortion as a way to rationalize or justify their abortions, but rather to better understand their experiences [23].

Denying women an abortion, which occurred among one quarter of the women interviewed in this study, may have a significant negative impact on her health, her existing children and other family members, and her future. Policies that restrict access to abortion must acknowledge that such women will need added support (e.g. financial, emotional, educational, health care, vocational support) to appropriately care for their children, other children, and themselves. In some cases, where women are struggling with abuse or health issues, continuing an unwanted pregnancy to term may be associated with even greater than normal risks of childbirth.

This study should be viewed in light of its limitations. Fewer than 40% of women who were eligible and approached agreed to participate. Many women may have been deterred from enrolling because participation required bi-annual interviews for a period of 5 years. Nonetheless, our sample demographics, with the exception of our overrepresentation of women beyond the first trimester, closely mirror the national estimates of women seeking abortion in the US, suggesting that our results are generalizable [24,25]. The greater proportion of women in our sample seeking abortions at later gestational ages and without fetal anomalies allows us to make inferences about a previously understudied group. Gestational age at the time of the interview was unrelated to any of the major themes mentioned. Other studies have found that late gestational age was an important predictor of termination because of concerns about the health of the fetus [9]. In this study, we have excluded women seeking abortion for fetal anomaly and found that seeking a later abortion was unrelated to women’s reasons for seeking an abortion. Thus, among women without fetal anomalies, reasons for seeking abortion are not different whether women sought abortion early or late in pregnancy. This suggests that factors other than the reasons for desiring an abortion play a role in seeking later abortions.

A small number of women stated that concern for the fetus while using contraception or other medications was a reason for seeking abortion pointing to an area for intervention. The general consensus in the literature is that birth control use during pregnancy is unlikely to have negative consequences for the development of the fetus [26-29]. A better understanding of the potential impact of the contraceptive methods and other medications on a developing fetus can help women be better informed when deciding whether nor not to have an abortion.

Laws requiring waiting periods, mandated counseling, and parental involvement for adolescents are motivated in part by a desire to protect women from making uninformed decisions and from being coerced into having an abortion. Prior research suggests that, women who feel the abortion decision is not completely their own have more difficulty coping following an abortion [30]. Our study, like most studies of women seeking abortions [9], finds that few women report pressure from others as a reason for seeking abortion. About 1% of women in this study described being influenced by others to have an abortion. Our study design, however, did not allow us to assess the level of pressure women experienced. The pressure women felt may have varied in degree from statements of a mild lack of support for continuing a pregnancy to strong and specific statements about a lack of future emotional or financial support for the pregnancy or potential child. While these women’s pregnancy intention scores are somewhat higher than those who gave other reasons for abortion, their scores were still in the unintended/ambivalent range. Health care providers should continue to assess and confirm that women are able to make their own decision about whether or not to continue or end a pregnancy. Women who experience pressure may benefit from additional emotional support if they choose to proceed with abortion.

In recent years, politicians, advocacy organizations and the media have extensively debated issues related to the funding, provision, utilization, and morality of abortion, and legislation restricting abortion access has increased dramatically. The Guttmacher Institute documented that 92 new provisions restricting abortion were enacted in 2011, almost three times the previous record of 34 provisions enacted six years earlier [31]. Despite the proliferation of proposed legislation that would restrict access to abortion, the public discourse concerning why women seek abortions has been limited. It is important that policy makers consider women’s motivations for choosing abortion, as decisions to support or oppose such legislation could have profound effects on the health, socioeconomic outcomes and life trajectories of women facing unwanted pregnancies.

## Conclusion

As found in previous literature, the findings from this study demonstrate that women are motivated to seek abortion for a wide range of reasons that are driven by their unique circumstances and stage of life. Women expressed lacking the financial, emotional, and physical resources to adequately provide for a/another child, yet many were denied access to a wanted abortion. Supporters of policies that continue to further restrict women’s access to abortion need to recognize the potential impact on the financial, emotional, and physical well-being of these women and their families. Women who carry an unwanted pregnancy to term because they are denied access to a wanted abortion may require financial assistance, support handling an abusive partner, access to mental health services prenatal care and, potentially, specialized health care for high risk pregnancies. By better understanding women’s decisions when faced with an unintended pregnancy and destigmatizing abortion seeking we can better support women’s reproductive decisions and provide them with the resources they may need.

## Endnote

^a^Bill of Rights can be downloaded at: http://www.research.ucsf.edu/chr/Guide/chrB_BoR.asp.

## Competing interests

The authors declare that they have no competing interests.

## Authors’ contributions

MAB’s role in this paper included conceptualizing the analyses for this paper, leading the quantitative and qualitative analyses and drafting the manuscript. HG was responsible for reviewing the literature, assisting in the qualitative coding, and drafting and editing the manuscript. DGF conceptualized and led the overall Turnaway study design and assisted in drafting and editing the manuscript. All authors approved the final manuscript.

## Pre-publication history

The pre-publication history for this paper can be accessed here:

http://www.biomedcentral.com/1472-6874/13/29/prepub
